# Surface characterization of maize-straw-derived biochar and their sorption mechanism for Pb^2+^ and methylene blue

**DOI:** 10.1371/journal.pone.0238105

**Published:** 2020-08-27

**Authors:** Chunbin Guo, Jingjing Zou, Jianlin Yang, Kehan Wang, Shiyu Song

**Affiliations:** 1 Department of Materials Science and Engineering, Liaoning Technical University, Fuxin, Liaoning, China; 2 Department of Environment Science and Engineering, Liaoning Technical University, Fuxin, Liaoning, China; Qatar University, QATAR

## Abstract

Biochar derived from straw is a potential low-cost adsorbent for metal ions and organic pollutants, but its practical application is still limited by the adsorption capacity. In this study, the correlation between the biochar’s properties and pyrolysis temperature was explored. The adsorption mechanism was studied by monitoring the changes of biochar properties before and after adsorption using BET, SEM, XPS and FT-IR spectroscopy. The adsorption mechanism was revealed following the adsorption kinetics and the changes in biochar’s properties before and after adsorption. The methylene blue (MB) and Pb^2+^ adsorption removal efficiency reached 95% at the initial concentration of 125 and 500 mg/L, respectively. Physisorption, chemisorption, and pore filling mechanisms determined the adsorption process of MB and Pb^2+^ on biochar. The Pb^2+^ adsorption process was highly affected by chemical co-precipitation at higher pyrolysis temperatures. The appearance of tar particles increased the adsorption rate of Pb^2+^. The biochar obtained at the pyrolysis temperature at 500, 800 and 900°C proved to be applicable for Pb^2+^ removal. Chemisorption and porosity dominated the MB adsorption, and biochars produced at pyrolysis temperatures of 200, 800 and 900°C are potential materials for MB removal. This study provides optimal pyrolysis conditions for transforming maize straw into valuable, low-cost materials for the removal of different pollutants.

## 1. Introduction

Biochar is widely used, porous carbonaceous material for the purification of water/wastewater and the remediation of contaminated soil [1–3]. Biochar is mainly obtained via pyrolysis of organic biomass under anoxic conditions [4–6]. With the development of slash-and-char technology, the pyrolysis of biomass is more extensively used for the production of biochar and bioenergy. Biochar represents a promising alternative to activated carbon due to the low cost [7–9]. The purification of soil and water depends on several properties of biochar such as surface area (SA), the abundance and type of functional groups on the surface and pore volume (PV) [10–12]. All these properties vary with the pyrolysis temperature, so the pyrolysis procedure should be optimized to obtain the appropriate biochar.

The comparative advantages of biochar are good adsorption properties toward pollutants [13], low price, and excellent availability. The binding energy between the biochar and pollutant depends on the chemical nature of both compounds. The adsorption capacity of biochar and the affinity for pollutants can be strongly affected by incorporating organic functional groups on the surface and/or inside the pores [14–16]. Therefore, it is very important to explore the mechanisms by which environmentally relevant pollutants such as metal ions and organic compounds are removed by biochar.

The nature of feedstock strongly affects the properties of biochars [17, 18]. For example, biochar derived from low-cost biomass resources such as grass, reed, and orange peel showed different sorption capacities for dyestuf [19–21]. As maize straw is highly abundant in China, it represents an important feedstock for biomass production [22–24]. Hence, it is important to select suitable pyrolytic conditions for maize straw.

In this research, the changes in physicochemical properties of maize straw biochar with the pyrolysis temperature were studied. These properties include micro-changes in structure, SA, PV, and functional groups on the surface. The changes in morphology and functional groups of biochar before and after the adsorption were described. The adsorption mechanism of biochar produced at different pyrolysis temperatures was studied in detail using dyestuff (MB) and heavy metal ion (Pb^2+^) as model compounds. The most suitable pyrolysis conditions, giving the biochar with the optimum removal characteristics and utilization of waste straw material, were provided for both pollutants.

## 2. Materials and methods

### 2.1 Biochar fabrication

**Fig 1:**
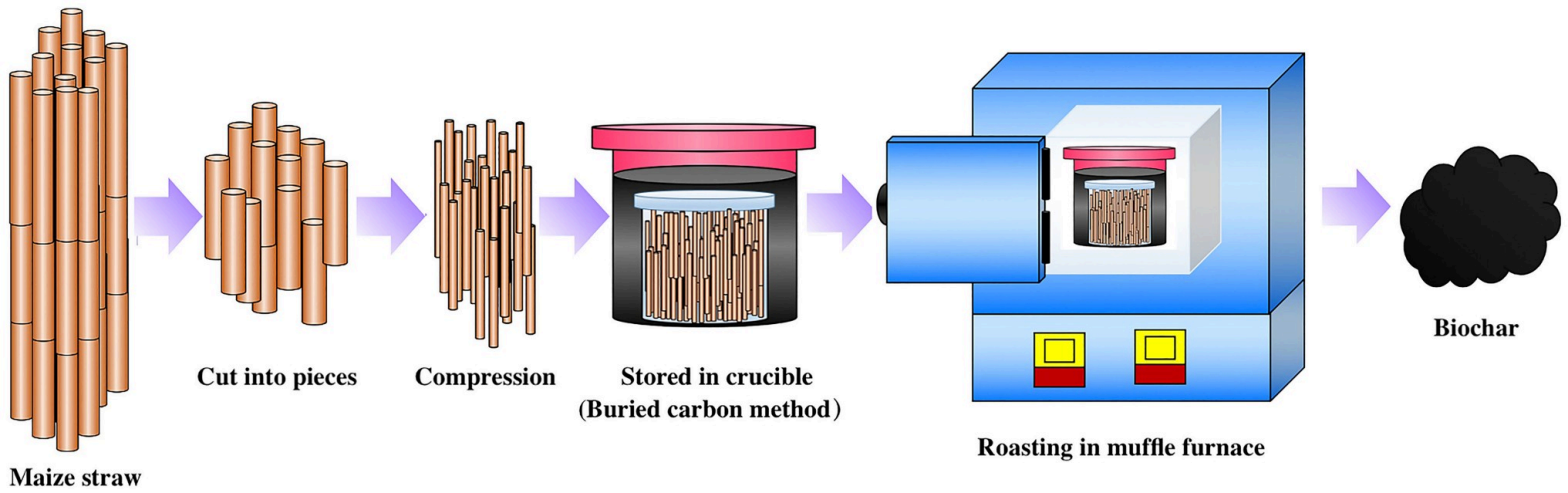
Process flow diagram of biochar preparation from maize straw.

Raw maize straw material was obtained from a plowland in Fuxin, Liaoning Province, China. In this area, maize is planted once per year and the growth cycle is longer compared to other places. Here, maize straws were burned as agricultural waste, no special permit is required to collect them. Maize straws were washed two times with water to remove the adsorbed impurities. The straws were dried in air for two days and stoved at 100 °C for 6 h in small crucibles. Afterward, the long-straws were cut into pieces (8–10 cm) and loaded into the larger crucibles. The charcoal powder was added to remove the oxygen. The flow diagram is illustrated in Fig 1. The straws were stored in crucibles and heated in a muffle furnace for 3 h at different pyrolysis temperatures. After cooling, the biochars were smashed and passed through a 60 mesh sieve (0.25 mm). In this study, the pyrolysis temperature ranged from 200 to 900 °C, and the produced biochars are labeled as S200, S300, S400, S500, S600, S700, S800, and S900. The chemical reagents used in all experiments were p.a. grade.

### 2.2 Characterization of biochar

Morphology of products and their acid residues and was visualized by scanning electron microscope (SEM). The cations in an aqueous solution were quantified by inductively coupled plasma (ICP) emission spectroscopy. The Zeta potential of biochar was determined by Micro electrophoresis device (JS94K2, China). The specific SA of biochar samples was determined by N_2_ adsorption analyzer. The surface functional groups of samples were analyzed using Fourier transform infrared (FTIR) spectroscopy and X-ray photoelectron spectroscopy (XPS).

### 2.3 Adsorption studies

To explore the effect of different pyrolysis temperature on heavy metal (Pb^2+^) and dyestuff (MB) adsorption on biochars, 0.05 g of biochar samples was mixed with 50 mL of MB and Pb^2+^ solutions in 100 mL conical flasks at 25°C. The metal solution (100–500 mg/L) was obtained by dissolving Pb(NO_3_)_2_ in deionized H_2_O. The dyestuff solution (2.5–10 mg/L) was obtained by solubilizing MB in deionized H_2_O. The pH was set to 3 using hydrochloric acid.

The biochar samples were placed in a solution and shaken at 120 rpm for 13 h to equilibrate, then passed through a 0.45 μm syringe filter, and an aliquot of the supernatant was collected for chemical analyses. The difference in concentration of the pollutant in a solution before and after the adsorption was used for calculating the amount of substance adsorbed per unit mass of biochar (*Q*). All measurements were baseline-corrected. All results are reported as a mean of two independent measurements.

### 2.4 Parameters of adsorption kinetics

The changes in *Q* with time were fitted into the pseudo-first and pseudo-second-order kinetic models to investigate the adsorption kinetics:

$$\text{ln}(q_{e}-q_{t})=\text{ln}q_{e}-K_{1}t$$ (1)

$$\frac{t}{q_{t}}=\frac{1}{K_{2}q_{e}{}^{2}}+\frac{1}{q_{e}}t$$ (2)

In these equations, *q*_t_ (mg/g) represents the amount of Pb^2+^ absorbed by biochar at time *t*. *K*_1_ (min^-1^) and *K*_2_ (g·mg^-1^·min^-1^) represent rate constants of the adsorption.

## 3. Results and discussion

### 3.1. Characteristics of maize straw biochars

#### 3.1.1 Morphology

**Fig 2:**
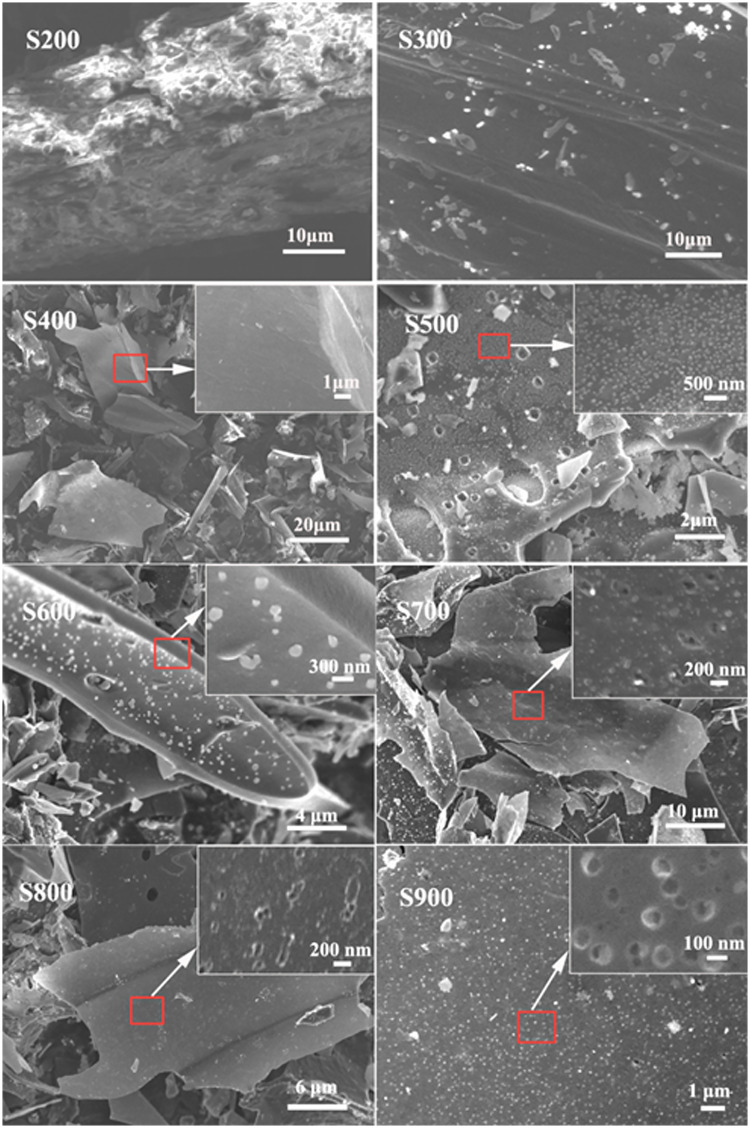
SEM images of biochar samples generated from 200 to 900 °C.

The surface morphology of maize straw-derived biochars is shown in Fig 2. The surface morphology of maize straw-derived biochar was strongly influenced by pyrolytic temperature. The results indicated three stages during the pyrolysis.

In the first stage (200–400°C), the hemicellulose, cellulose, and lignin converted to volatile matter and charcoal, resulting in the break-up of large pieces into many small, irregular fragments. For biochar S200, samples retained the surface structure of straw material. In line with this, lower electrical conductivity indicated incomplete carbonization. Increasing the pyrolysis temperature up to 400°C, the surface of samples became smooth and electrical conductivity increases.

In the second stage (400–600°C), the increase in pyrolysis temperature to 500°C induced the formation of a large number of macropores (diameter >300 nm) on the surface of biochar. Meanwhile, many irregular particles (wood tar, diameter <100 nm) coexisted on the surface of biochar due to the loss of aliphatic functional groups. Further increase in temperature resulted in a formation of more pores, lowered the number of wood tar particles, and smoothed the surface of biochar particles.

In a third stage (700–900°C), bright points occurred on the surface of biochar. Further observations revealed that the bright points originate from micropores that substituted the wood tar. As can be seen from S700 to S900, the increasing number of micropores was forming, and the diameter of biochar was decreasing, suggesting that these biochars might possess a fine microporous structure.

#### 3.1.2 The functional groups on the surface of biochars

**Fig 3:**
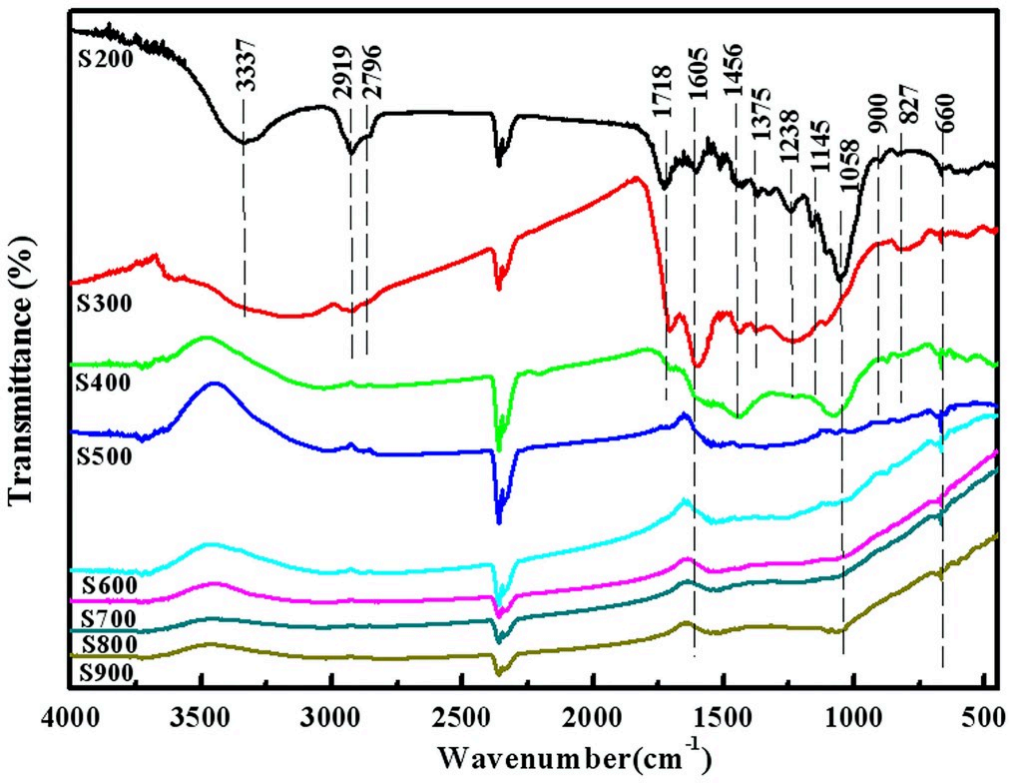
FTIR spectra of biochar samples generated from 200 to 900 °C.

The changes in the type and number of functional groups on the surface of biochars were analyzed by FTIR, Fig 3. In the case of S200, spectral bands at 3337 cm^-1^ that correspond to–OH groups were profound. The spectral bands at 2919, 2796, 1718, 1605, 1375, 1238, 1145, 1058, 909 and 827 cm^-1^ were assigned to asymmetrical stretching vibration of C–H_2_ [20, 24, 25], symmetric stretching vibration of C–H, C = O, aromatic, C–H_3_, C–O, C–O–C, C = C and C–H [26, 27], respectively. The symmetric stretching vibration of C–O moved to higher wavenumber with an increase in the pyrolysis temperature. In the case of the biochar produced at 500°C, almost all of the aliphatic surface functional groups disappeared/weakened. Along with this, the content of aromatic surface groups (aromatic C = O, aromatic C = C, and aromatic CO–) remained nearly unchanged compared to lower pyrolysis temperatures. Further increase in the temperature of pyrolysis induced the appearance of Si-O spectral band (around 600 cm^-1^) [25], indicating the higher portion of carbonized biochar.

#### 3.1.3 Specific surface area (SA) and pore volume (PV)

**Fig 4:**
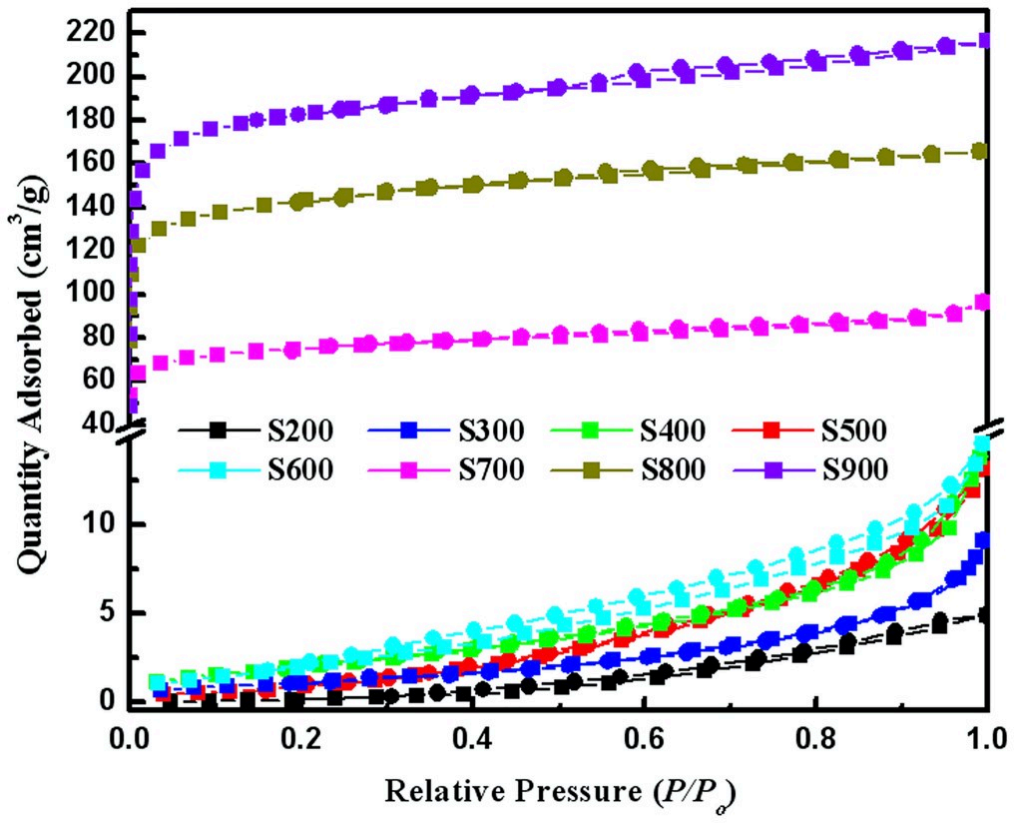
Adsorption–desorption isotherm of samples generated from 200 to 900 °C.

The pore structure (including PV distribution and specific SA, and pore size) of biochar plays a key role in the adsorption performance. The polar functional groups were gradually removed with an increase in pyrolysis temperature. A strong correlation between pyrolysis temperature and SA of the resulting biochar was observed. The pore structure of biochar samples was analyzed by N_2_ adsorption/desorption method (Fig 4 and Table 1). The trends in curves representing the volume of N_2_ adsorbed by biochar samples are significantly changed as temperature increases.

**Table 1:** Pore volume, surface area, for the samples generated from 200 to 900 °C.

Sample	S_BET_ (m^2^/g)	Smes (m^2^/g)	Vt (cm^3^/g)	Vmic (cm^3^/g)	Dp (nm)	Zeta
S200	0.473	0.003	0.008	0.000	0.009	-3.2
S300	3.093	0.002	0.020	0.000	15.403	-17.4
S400	3.773	0.002	0.014	0.000	13.085	-30.2
S500	6.904	0.009	0.021	0.001	10.861	-48.2
S600	6.926	0.016	0.023	0.003	9.975	-50.1
S700	261.602	182.702	0.149	0.092	2.503	-51.8
S800	497.835	344.970	0.256	0.180	2.333	-55.4
S900	635.244	464.289	0.335	0.231	2.292	-57.7

Vt = total pore volume, Vmic = micropore volume, S_BET_ = BET surface area, Smeso = mesopore surface area, Dp = average pore diameter

When biochar samples were produced at pyrolytic temperatures from 700°C to 900°C, the sudden increase in a volume of N_2_ adsorbed was observed from the isotherm at low relative pressures (P/P_o_), suggesting the Type I adsorption curve. The adsorption curve of S700 does not display an apparent desorption hysteresis loop, implying that the biochar samples are highly microporous. The H4-type hysteresis loops were observed for S800 and S900, indicating the existence of micropores and mesopores in the samples, where micropores were mainly slit-like [26].

When biochar samples were produced at lower pyrolytic temperatures (from 200 to 600°C), the isotherms display bulges in the opposite direction of pressure axis, suggesting the Type III adsorption curve. With the increase in pyrolytic temperature the hysteresis loops appeared, reflecting the existence of macropores or interstitial pores. The sharp rise in the quantity of N_2_ adsorbed near the P/P_o_ of 0.8–1.0 indicates the appearance of macropores in biochar samples.

Total PV and S_BET_ for biochars obtained from pine wood shavings or orange peels increased with temperature in the range from 500 to 700 °C. However, in the case of maize straw derived biochars, PV and S_BET_ kept increasing until 700 °C. At the pyrolytic temperature of 900 °C, the SA increased to 635 m^2^/g, and PV added up to 0.231 cm^3^/g. There are almost no holes in the low-temperature-pyrolysis straw (S200). The average pore diameter of biochar samples significantly decreased with the increase in pyrolysis temperature from 300°C to 600°C. Further increase in pyrolysis temperature to 700°C caused a sudden drop in the average pore diameter from 9.975 nm to 2.503 nm, suggesting the formation of a large number of micropores. The high BET surface and micropore areas of S800 and S900 suggested a fine porous structure, which is in agreement with SEM results (Refer to SEM images). The Zeta potential rapidly increased with the increase in pyrolysis temperature from 200°C to 500°C (Table 1), as well-carbonized biochar has larger Zeta [19–21].

**Fig 5:**
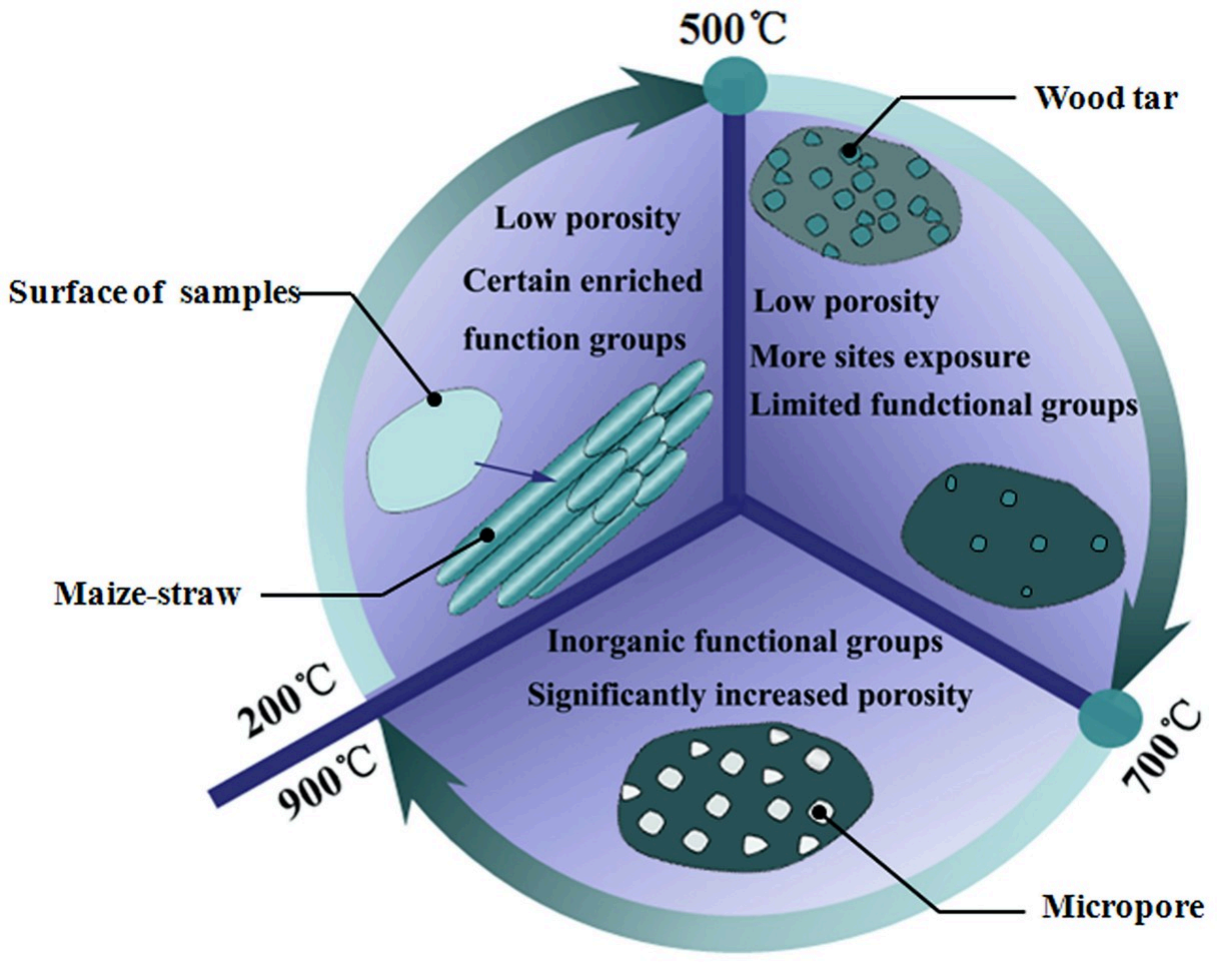
Schematic diagrams of biochar properties at different temperatures.

The influence of different pyrolysis temperatures on biochars is summarized in Fig 5. The total PV and S_BET_ rapidly increased during the temperature rise from 700°C to 900°C. As temperature increases from 200°C to 500°C, organic functional groups slowly deplete from the biochar and gather into small particles (wood tar) on the surface of biochar. From 500°C to 700°C, the amount of small particles is reduced and a larger number of active sites for adsorption are exposed.

### 3.2 Evaluation of the adsorption capacity for pollutants

#### 3.2.1 Sorption performance of biochars

**Fig 6:**
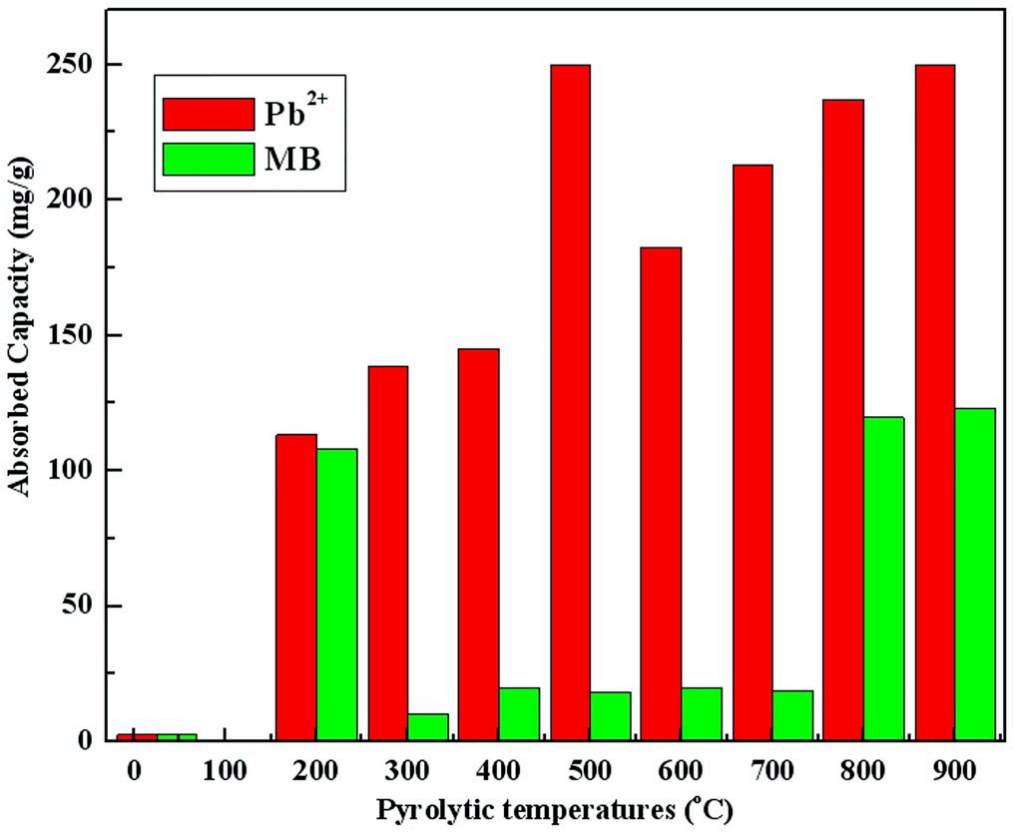
Effect of biochar pyrolytic temperatures on the adsorption capacity of Pb^2+^ and MB.

To investigate the effect of different pyrolysis temperatures on Pb^2+^ and MB adsorption, 0.05 g of biochar samples was used as sorbent. The sorbents were mixed with 50 mL of MB (125 mg/L) and Pb^2+^ (250 mg/L) solutions. All experiments were performed in triplicates. Fig 6 illustrates the removal of Pb^2+^ and MB for biochar after static sorption for 13 h. The significant difference in Pb^2+^ and MB adsorption capacity on biochar was observed.

The results revealed a sudden increase of MB adsorption capacity of biochar produced at 200 and 800°C (S200 and S800). The trend in MB adsorption capacity corresponded to PV and BET results for all samples except S200, suggesting that specific SA mainly drives the adsorption process of MB on biochar. As already shown by FTIR, the surface functional groups of S200 are different from other samples (C–H_2_, C = O, C–H_3_, C–O, C–O–C, C = C, and C–H) which probably influences the adsorption of MB. This adsorption process is more inclined to chemical bonding. Therefore, S200 has the strong adsorption capacity. When the Zeta potential is in the range of 0 to -50, the adsorption capacity of biochar to MB basically remains unchanged or changes very little, and the electrostatic attraction of MB and biochar surface can be slightly neglected, so the adsorption phenomenon is mainly caused by the van der Waals attraction between them. When the Zeta potential is less than -50 mV, the negative potential on the activated carbon table plays a leading role in the electrostatic attraction of cationic MB, which makes the adsorption increase sharply Therefore, MB adsorption capacity of S800 and S900 increased sharply.

All of the biochars (S200-S900) showed a good performance in Pb^2+^ adsorption. The Pb^2+^ adsorption capacity of biochar gradually increased with the increase in pyrolysis temperature for all samples except S500. The Pb^2+^ adsorption capacity of biochar increases with the increase of Zeta potential, which may be due to the dominant role of electrostatic attractor between lead ion and biochar. The adsorption capacity increased suddenly of S500 might be explained by SEM, where S500 sample showed a larger amount of wood tar compared to other biochars (add cross-reference to SEM image), suggesting the important role of irregular particles on the surface of biochar for Pb^2+^ adsorption. The irregular particles consist of a larger amount of C-O and C = O groups, which can coordinate Pb^2+^ ion forming C-O-Pb. Going from S200 to S500, the number of irregular particles increased which improved the Pb^2+^ adsorption capacity. Going from S600 to S900, the number of irregular particles decreased, but Pb^2+^ adsorption capacity still increased gradually. According to BET results, we assumed that the higher SA compensate for the decreased number of irregular particles, resulting in the increased Pb^2+^ adsorption capacity. Therefore, the Pb^2+^ adsorption properties of biochar depend on the presence of irregular particles and specific SA.

**Fig 7:**
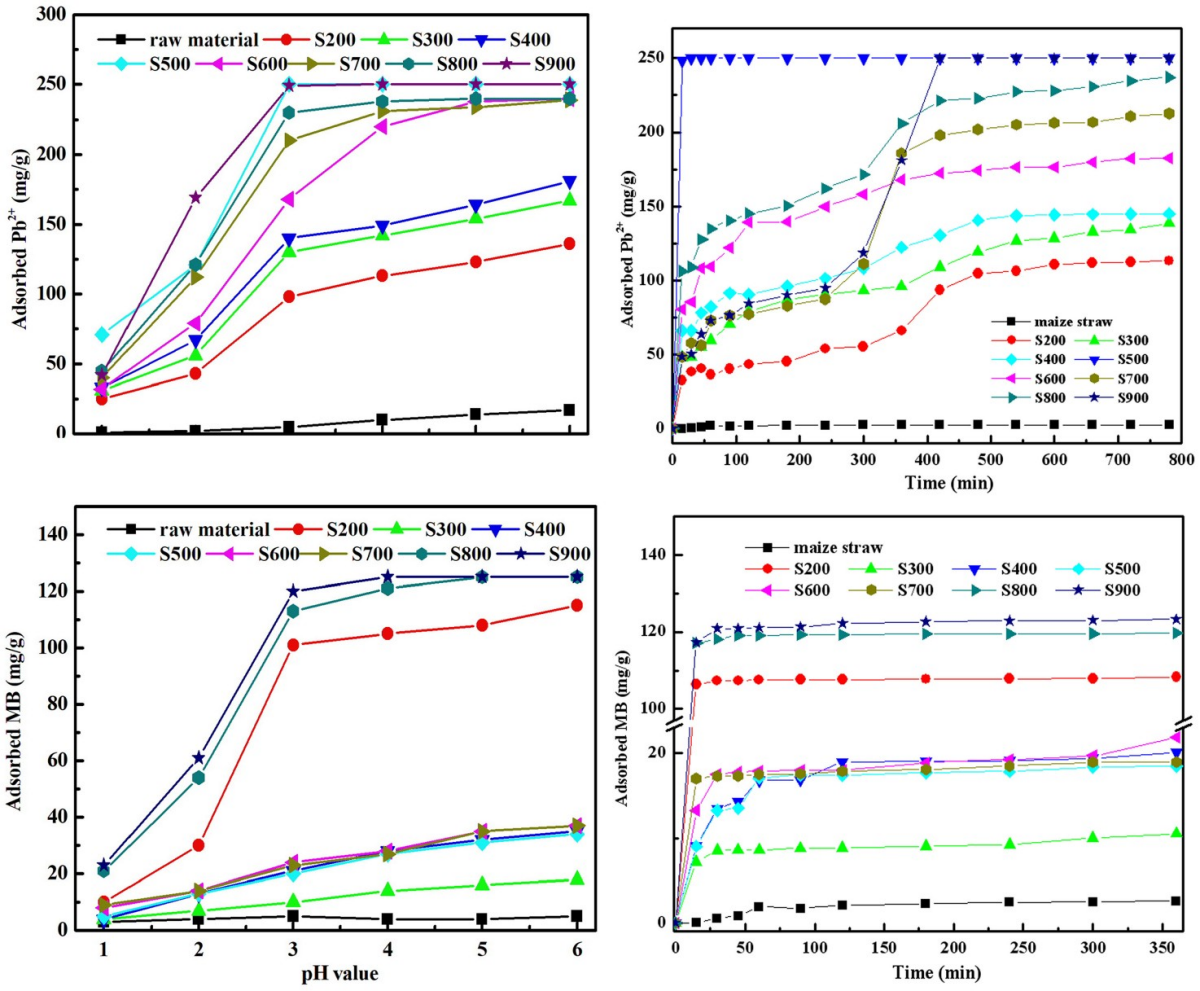
The variation of adsorption uptake of Pb^2+^ and MB with time by biochar samples derived from different pyrolytic temperatures: (a) Pb^2+^ (b)MB.

The effect of contact time and pH value on Pb^2+^ and MB uptake by biochar with respect to temperature was presented in Fig 7. The pH of the aqueous system largely affects adsorption of Pb^2+^ and MB. As can be seen from the Fig 7, the MB adsorption capacity increased as the solution pH increased from 1 to 6. The MB adsorption capacity of S200, S800 and S900m were rapid increased at pH = 3, which might due to the different dominant sorption mechanisms on these sorbents. Surface mulching is the main surface adsorption mechanism of biochar at high-temperature carbonized. There are other surface adsorption mechanisms of biochar at high-temperature carbonized, such as multilayer tiling, capillary phenomenon or inner pore filling. The Pb^2+^ adsorption capacity fast increased from solution pH 1 to 3. The adsorption capacity of biochar at low temperature carbonized grow slowly at solution pH 3 to 6. The carbonized straw at high temperature can adsorb more Pb^2+^ at low pH, which might due to surface mulching dominant sorption process.

The rapid adsorption of MB was observed in the first 1 h, after which the adsorption rate decreased until reaching the equilibrium. The adsorption rate of MB increased at higher temperatures for all samples except S200. The adsorption rate of Pb^2+^ could be divided into three parts. The rapid adsorption of Pb^2+^ was observed in the first 1 h, and the adsorption rate decreased until 5 h. This might be explained by the film and pore diffusion as the rate-limiting parameter in the first 1 h, while the decrease in Pb^2+^ adsorption rate is due to the saturation of adsorption sites on biochar’s surface. In the third stage, the adsorption rate of Pb^2+^ suddenly increased between 5 h to 7 h, after which the adsorption rate slows down until reaching an equilibrium. This process might be explained by chemical co-precipitation.

#### 3.2.2 Kinetic study

**Fig 8:**
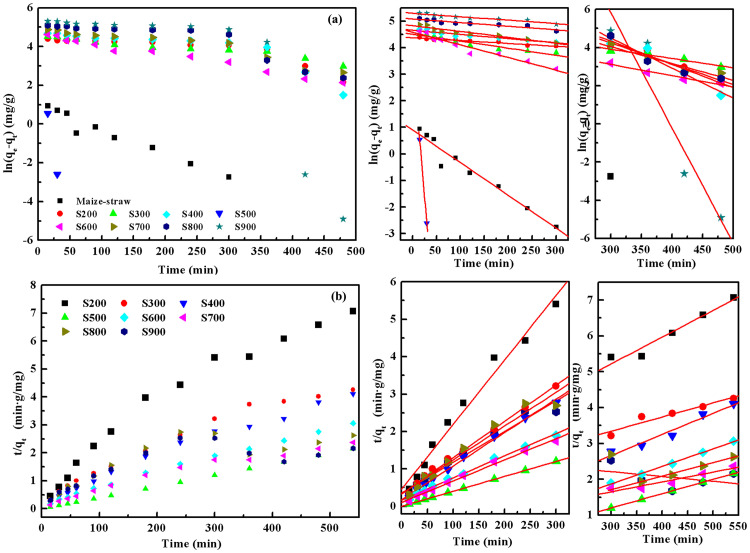
The kinetic model fitted to experimental data for Pb^2+^ removal by biochars: (a) Pseudo-first-order (b) Pseudo-second-order.

The data for Pb^2+^ and MB adsorption by biochar were simulated using pseudo-first and pseudo-second-order kinetic models in order to investigate the adsorption kinetics (Table 2 and Fig 8).

**Table 2:** Comparison of pseudo-first-order and pseudo-second-order kinetic model rate constants calculated from experimental data.

Sample	Pollutant	Pseudo-first-order						Pseudo-second-order					
		q_e_ (mg/g)		K_1 *_10^−3^ (min^-1^)		R^2^		q_e_ (mg/g)		K_2 *_10^−3^ (g/mg.min)		R^2^	
S200	MB	12.3		3.97		0.962		46.3		0.81		0.979	
S300	MB	23.8		2.55		0.957		83.8		0.33		0.993	
S400	MB	29.2		10.01		0.826		89.2		0.56		0.995	
S500	MB	68.7		8.22		0.828		200		166.7		1	
S600	MB	52.6		2.54		0.972		82.8		0.27		0.995	
S700	MB	57.4		6.62		0.955		91.4		0.37		0.994	
S800	MB	74.3		7.86		0.847		134.4		0.4		0.983	
S900	MB	90.1		9.3		0.981		140.8		0.3		0.986	
Sample	Pollutant	Pseudo-first-order						Pseudo-second-order					
		q_e1_	K_1 *_10^−3^	R_1_^2^	q_e2_	K_2 *_10^−3^	R_2_^2^	q_e3_	K_3 *_10^−3^	R_3_^2^	q_e4_	K_4 *_10^−3^	R_4_^2^
S200	Pb^2+^	2.5	1.1	0.93	1866.7	10.9	0.95	57.84	0.65	0.980	134.05	0.02	0.96
S300	Pb^2+^	79.8	2.7	0.93	215.11	4.9	0.93	103.09	0.28	0.994	253.81	0.01	0.93
S400	Pb^2+^	39.5	1.6	0.91	9931.3	15.7	0.94	111.48	0.45	0.995	169.49	0.04	0.96
S600	Pb^2+^	105.9	4.8	0.95	133.1	5.9	0.96	168.07	0.22	0.995	204.08	0.06	1.00
S700	Pb^2+^	98.3	2.2	0.94	794.5	8.8	0.92	176.06	0.3	0.995	362.32	0.01	0.92
S800	Pb^2+^	125.6	1.4	0.89	2984	12.2	0.91	110.38	0.25	0.958	1910.59	0.01	0.91
S900	Pb^2+^	161.4	1.3	0.93	9885	60.3	0.91	121.51	0.19	0.965	7462.69	0.01	0.40

As can be seen from the correlation coefficients (R^2^) of the adsorption data for MB, both pseudo-first and pseudo-second-order kinetic models have high fitting degree, the R^2^ of pseudo-second-order model was slightly higher than pseudo-first-order model. It indicates that the adsorption rates toward MB were controlled by chemical interactions.

The adsorption process of Pb^2+^ is stepped over time, the kinetic discussion is divided into two parts. The experimental data for Pb^2+^ adsorption also fitted the pseudo-second-order model (higher R^2^ values), indicating chemical interactions as a controlling factor for Pb^2+^ adsorption on biochar. Furthermore, the R^2^ of S800 and S900 was lower than others, this indicating he adsorption mechanism changes, especially in the second half of the adsorption process. Two distinct linear parts were observed in the Fig 8. In the first stage, rate constant *k*_1_ was higher than the rate constant of subsequent step, *k*_2_. This result indicates the change of a dominant adsorption mechanism from adsorption to chemical co-precipitation, where the first stage is faster than the second one.

By analysing the samples at different pyrolysis temperatures, it can be seen that chemical precipitation occurs earlier and the faster Pb^2+^ removal rate of S800 and S900, this also indicates the high BET, PV and low Zeta potential can significantly change the adsorption mechanism, and promote the formation of chemical precipitation and improve the removal efficiency of lead ions.

#### 3.2.3 Adsorption mechanisms

**Fig 9:**
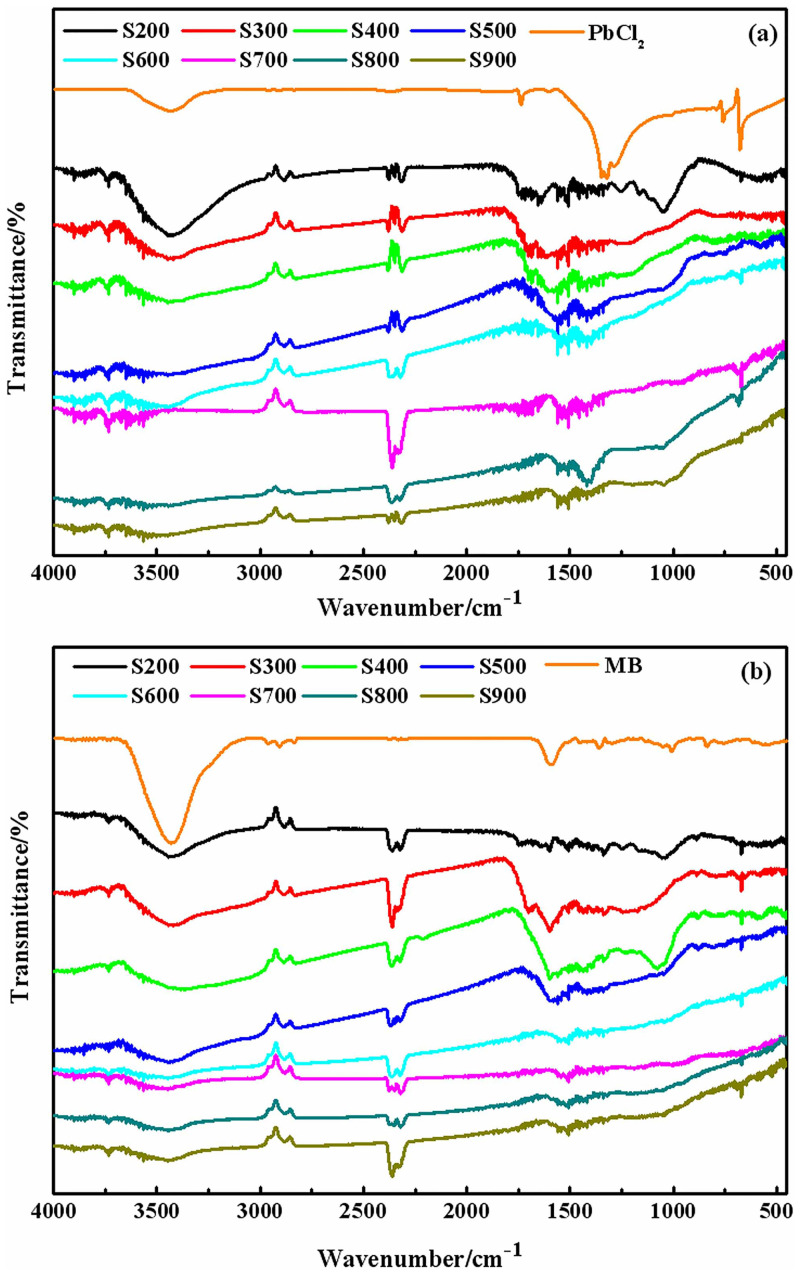
FTIR spectra of biochars after adsorption Pb^2+^ and MB. (a) Pb^2+^ (b) MB.

To further explore the adsorption mechanism, FTIR, SEM and XPS analysis of biochar were performed after sorption. The changes in functional groups on the biochar surface upon adsorption of Pb^2+^ and MB are illustrated in Fig 9. Compared with Fig 3, the symmetric stretching vibration peaks of C–O, C–O–C, C = C in S200 were moved to lower wavenumber, and the intensity of these peaks decreased. This indicates that the sorption of MB on S200 was mainly determined by the small percentage of carbonized surface and high yield of amorphous organic material. Increasing the pyrolysis temperature, the number of non-carbonized organic functional groups (C–O, C–O–C and C = C) decrease, and tar particles form on the surface of biochar. Hydrophilic functionalities on the surface of biochar interact strongly with the water molecules forming a cluster. This modifies the polarity of the surface and hinders the adsorption sites, reducing the adsorption rate. This explains the higher adsorption capacity of S200 than S300 and S400. When the pyrolysis temperature is above 700°C, a large number of pores appear and the tar particles disappear, leading to the increase in the number of sorption sites. As a result, the adsorption capacity of MB is improved.

As can be seen from Fig 9b, the intensity of organic functional groups (C–O, C–O–C and C = C) decreased, and the change is much smaller than the change given in Fig 9a. This suggested that the sorption mechanism of Pb^2+^ to biochar was not related to non-carbonized organic matters, but the properties of the high-carbonized material and abundant amorphous organic matter determined the Pb^2+^ adsorption. Furthermore, hydrophilic groups on the surface of biochar form water clusters and hinder the adsorption sites. Therefore, biochars synthesized at higher pyrolysis temperatures showed higher sorption capacities of Pb^2+^.

**Fig 10:**
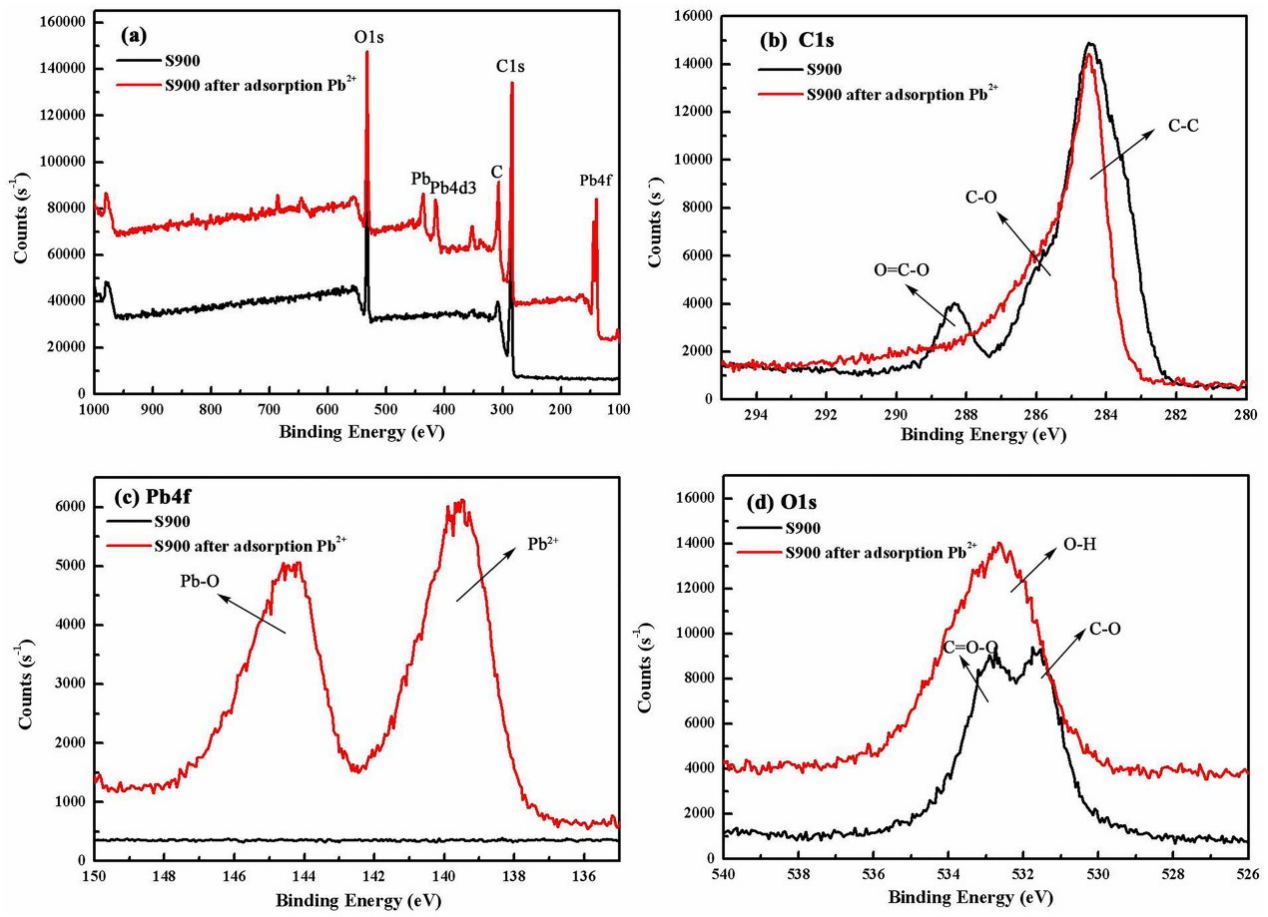
The XPS characterizations were performed on biochar before and after adsorption Pb^2+^.

To study the evolution in the binding energy and the surrounding chemical environment of a given atom, the XPS characterization was performed on biochar before and after the adsorption of Pb^2+^. The survey curves of C1s, Pb4f and O1s are presented in Fig 10. From Fig 10, the peaks at 284.6 eV, 285.9 eV and 288.7 eV are ascribed to the binding energy of C-C, C-O and O = C-O groups [11, 20]. The peaks at 531.36 eV, 532 eV and 532.51 eV were observed and assigned to C-O, O-H and C = O-O, and the peaks at 139 eV and 141.7 eV were assigned to Pb^2+^ and Pb-O respectively. After the adsorption of Pb^2+^, the binding energies of these peaks showed a slight shift. Fig 10a shows that the ratio of O = C-O and C-O in S900 decreases upon the adsorption of Pb(II). The changes indicated that a significant portion of hydroxyl and carboxyl groups participate in the adsorption process. Fig 10b and 10c show a significant increase in the peak area of O-H and Pb-O in S900 upon the adsorption of Pb^2+^. These changes illustrated that a large amount of Pb-O-H was generated during the adsorption process. Furthermore, the peaks of Pb^2+^ and Pb-O were observed in the S900 upon adsorption, showing that the process is governed by both physical and chemical adsorption.

**Fig 11:**
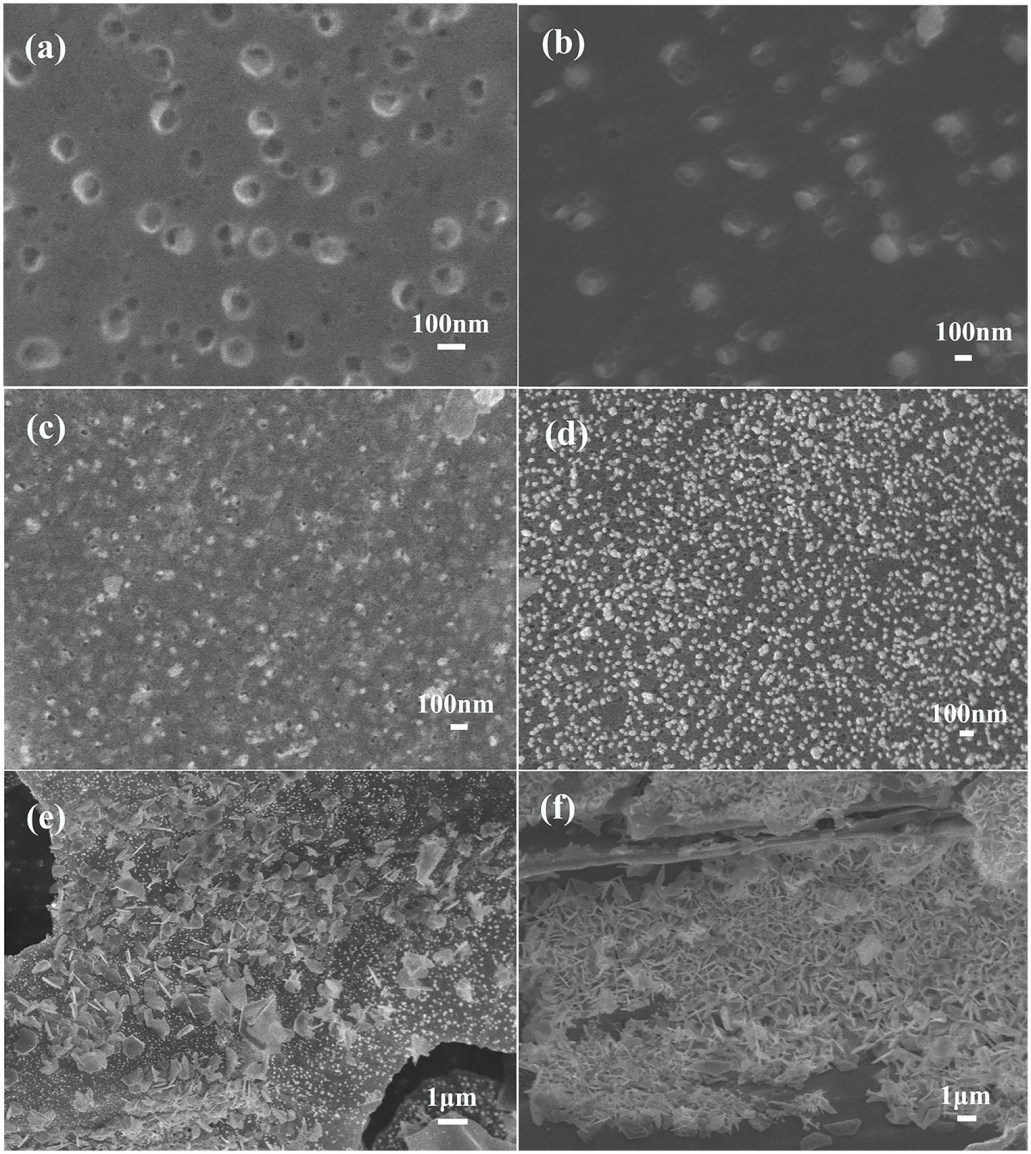
SEM images of S900 after adsorption different amount of Pb^2+^: (a) 48.5mg/g (b) 73.1mg/g (c)90.1mg/g (d)118.7mg/g (e)181.1mg/g (f)249.9 mg/g.

To additionally explore the reduction-adsorption process of Pb^2+^, the morphology of S900 after the adsorption of Pb^2+^ was tested (Fig 11). The results demonstrated that the adsorption process is divided into three stages. The microporous structure of biochar provides a large number of adsorption sites for Pb^2+^. In the first stage, biochar pores are occupied by Pb^2+^ but not completely blocked, as indicated by the high volume of micropores. This result corroborates the pore-filling adsorption mechanism. In the second stage, Pb^2+^ ions adsorb on the biochar surface and aggregate into the small particles. In the first stage, when pores are less occupied, the adsorption process is diffusion-limited, but the adsorption rate decreases with time as sites become more occupied and finally saturated by Pb^2+^. In the third stage, the increasing number of sheets is stacked on the surface of the biochar until the entire surface was covered. In this stage, the effect of chemical co-precipitation gradually increased. This effect increases the Pb^2+^ removal rate and the adsorption of Pb^2+^ gives a two-step curve. During the saturation of active site pores through the Pb^2+^ adsorption, the pH value of the solution gradually increased. Biochars produced at higher pyrolysis temperatures have larger PV, S_BET_ and the number of adsorption sites, providing better mass transport of the pollutants. Also, the onset time of chemical precipitation was improved. Therefore, two sheets are most likely generated during adsorption.

## 4. Conclusion

The adsorption mechanism of MB comprises of two stages–initial, diffusion-limited step, and the final step driven by adsorption forces. Moreover, the adsorption of MB depends on different types of interactions such as physisorption, chemisorption and pore filling. The pyrolysis temperature of 200, 800 and 900°C provided biochar with good adsorption capacity for MB. Chemical adsorption is a dominant process for S200. The increase in total PV, S_BET_ and the number of active sites improved the adsorption capacity of biochar. At the pyrolysis temperatures above 700°C, both physisorption and chemisorption play an important role. Semi-carbonized organic functional groups (irregular particles on the surface of biochar) have little influence on the MB adsorption capacity.

The transport via diffusion to surface/pores followed by chemical precipitation is the driving force for the adsorption process of Pb^2+^ on biochars. The strong adsorption of Pb^2+^ on biochar was explained by physisorption, chemisorption and chemical co-precipitation. Oppositely to MB, semi-carbonized organic functional groups (irregular particles on the surface of biochar) strongly influenced the adsorption capacity of biochar toward Pb^2+^ and increased the adsorption rate. With the increase in pyrolysis temperature, the chemical precipitation on biochar improved.

## Supporting information

S1 Graphical abstract . (JPG)
