# Correction: Surface characterization of maize-straw-derived biochar and their sorption mechanism for Pb^2+^ and methylene blue

**DOI:** 10.1371/journal.pone.0244938

**Published:** 2020-12-31

**Authors:** Chunbin Guo, Jingjing Zou, Jianlin Yang, Kehan Wang, Shiyu Song

Information is missing from the Funding statement. The correct Funding statement is as follows: This study was supported by Liaoning Provincial College Basic Research Program of Liaoning Education Department in the form of grants awarded to CG (LJ2017QL004) and JZ (LJ2017QL016, LJ2019FL001), the Industrial Public Relations and Industrialization Guidance Plan of Liaoning Provincial Department of Science and Technology in the form of grants awarded to JZ (20180551120) and CG (2019JH8/10100092), and The National Natural Science Foundation of China in the form of a grant awarded to JY (51404136). The funders had no role in study design, data collection and analysis, decision to publish, or preparation of the manuscript.
