# Supplementary figures and images for: Surface characterization of maize-straw-derived biochar and their sorption mechanism for Pb2+ and methylene blue
Source: PLoS One. 2020 Aug 27;15(8):e0238105. doi: 10.1371/journal.pone.0238105 (PMC7451984; doi:10.1371/journal.pone.0238105)

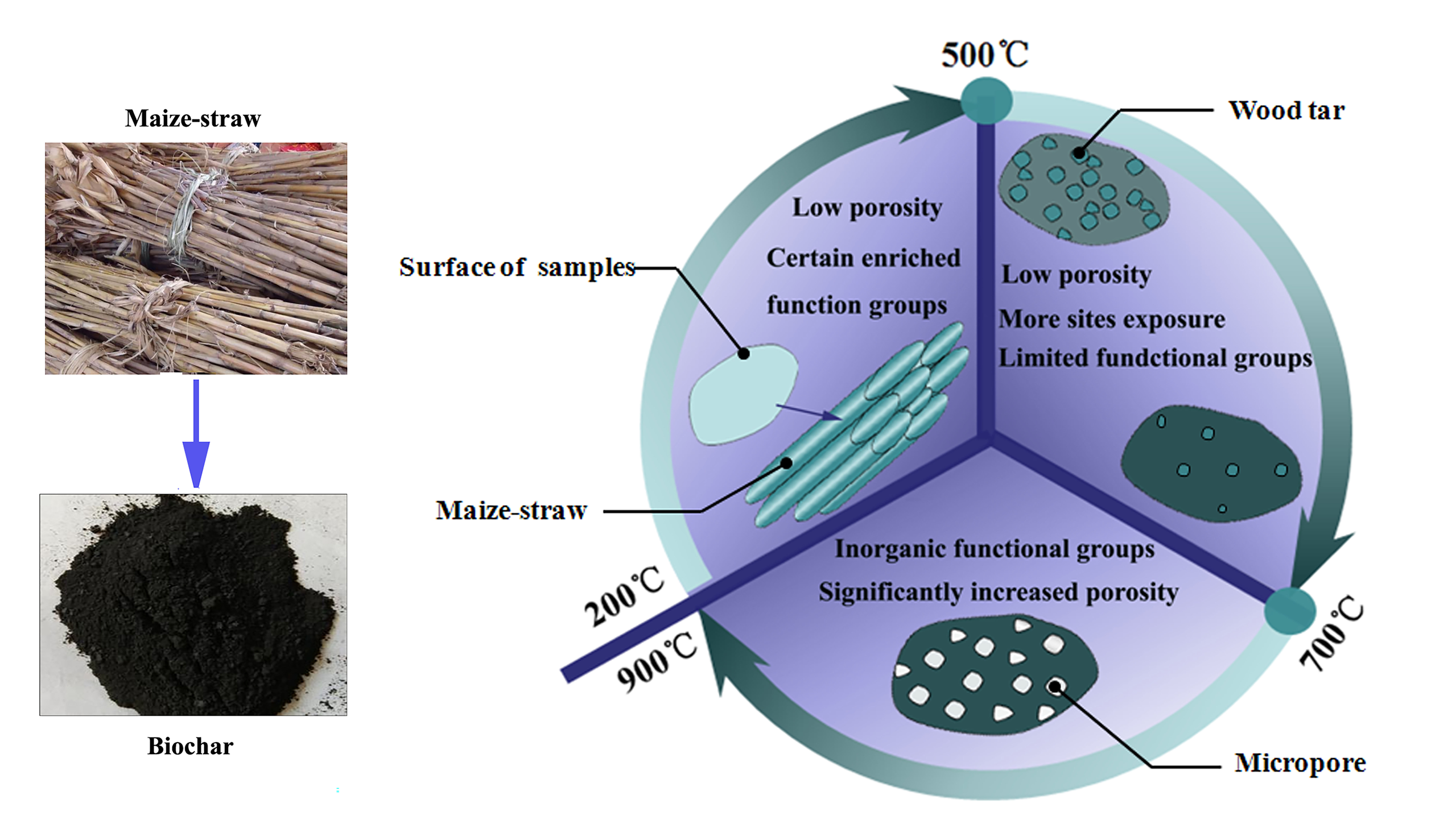

Supplement: S1 Graphical abstract — (JPG) [file pone.0238105.s001.jpg]
